# Plasma Polymerization of SnO*x*C*y* Organic-Like Films and Grafted PNIPAAm Composite Hydrogel with Nanogold Particles for Promotion of Thermal Resistive Properties

**DOI:** 10.3390/mi8010005

**Published:** 2016-12-26

**Authors:** Chin-Yen Chou, Ko-Shao Chen, Win-Li Lin, Ying-Cian Ye, Shu-Chuan Liao

**Affiliations:** 1 Department of Materials Engineering, Tatung University, Taipei 104, Taiwan; jasonno13@yahoo.com.tw (C.-Y.C.); kschen@ttu.edu.tw (K.-S.C.); 2 Institute of Biomedical Engineering, National Taiwan University, Taipei 106, Taiwan; winli@ntu.edu.tw; 3 Bachelor Program for Design and Materials for Medical Equipment and Devices, Da Yeh University, Changhua 515, Taiwan; an501290294@gmail.com

**Keywords:** AuNPs, NIPAAm hydrogel, plasma treatment, UV grafting

## Abstract

In this study, a new type of temperature sensor device was developed. The circular electrode of the thermally sensitive sensor was modified with tetramethyltin (TMT) and O_2_ plasma to form a thin SnO*x*C*y* conductive layer on the electrode surface. The nano-Au particles (AuNPs) were subjected to O_2_ plasma pretreatment to form peroxide groups on the surface. The thermally sensitive sensor made by mixing the treated AuNPs with N-isopropylacrylamide (NIPAAm) solution and then applying UV-induced grafting polymerization of the NIPAAm-containing solution onto the electrode substrate. The composite hydrogels on the electrode introduce thermo-sensitive polymeric surface films for temperature sensing. Using the ambient environment resistance test to measure the resistance, the lower critical solution temperature (LCST) of AuNPs mixed with NIPAAm hydrogel was found to be 32 °C. In common metallic materials, the resistance increased during environmental temperature enhancement. In this study, at ambient temperatures higher than the LCST, the electrode resistance decreases linearly due to the shrinkage structure with AuNPs contacting the circuit electrode.

## 1. Introduction

There are many types of commercially available temperature sensor elements such as thermistors, resistance-type temperature sensors, thermal couples and temperature sensing, etc. The advantages of resistance-type temperature sensors are high linearity, wide linear range, and a high output signal level and accuracy, but the disadvantage is the necessity to have three or four wired circuits, bulky components, and slow conduction and response. Thus, it is not suitable for rapid temperature measurement as well as for smaller objects.

Polymerized-N-isopropylacrylamide (P(NIPAAm)) is a well-known thermo-responsive polymer and exhibits a lower critical solution temperature (LCST) of about 32 °C in an aqueous medium. It assumes a random coil structure (hydrophilic state) below the LCST and a collapsed globular structure (hydrophobic state) above the LCST [1]. Using UV light, Peng and Cheng (1998) [2] grafted NIPAAm onto a porous polyethylene membrane. They noted that the temperature-responsive behavior of the grafted membranes changes with the grafting level. NIPAAm was also successfully grafted onto of many polymers for example: polyethylene terephthalate (PET), polypropylene (PP), poly(vinylidene fluoride) [3].

Because of the reversible phase transition, P(NIPAAm) has been used in the synthesis of thermally sensitive hydrogels for many applications [3,4,5,6]. P(NIPAAm)-based hydrogels absorb water and exist in swollen states below the LCST. However, they undergo an abrupt and drastic shrinkage in volume as the medium temperature is raised above the LCST. These unique characteristics allowed the P(NIPAAm)-based hydrogels to be used in biomedical applications, such as in the controlled release of drugs and in tissue engineering. Furthermore, it phase transition behavior can be controlled by incorporating a more hydrophilic or hydrophobic monomer in the gel composition [7].

It is well known that plasma treatment can alter the physical-chemical properties of a polymer surface. Improvements such as wettability, permeability, conductivity, printability, adhesion or biocompatibility can be easily achieved using plasma treatment in a very short time. The main reactions during plasma treatment on a polymer surface are etching, cleaning, crosslinking, grafting and other chemical reactions depending on the presence of active species in plasma [8]. The plasma deposition of tetramethyltin (TMT, Sn(CH_3_)_4_) has been found to be conductive to materials with useful antistatic coatings. This is because of their interesting gas sensing properties that have been widely applied to thin and thick film devices, flat display devices, transparent electrodes, anti-static films, thin-film resisters and heat reflectors [9,10,11,12,13,14,15].

The nano-Au particles (AuNPs) are suspension (or colloid) of sub-micrometer-sized particles of gold in a fluid usually water. The liquid is usually either an intense red color (for particles less than 100 nm), or a dark yellowish color (for larger particles). Due to the unique optical, electronic, and molecular-recognition properties of gold nanoparticles, they are the subject of substantial research, with applications in a wide variety of areas, including electron microscopy, electronics, nanotechnology, and materials science [16,17].

In this study, a novel thermal sensor device was successfully produced by combining the multi-properties of AuNPs and thermally sensitive P(NIPAAm) hydrogel. In addition, the plasma deposition technique was used for providing a conductive thin film on the circuit electrode. The resistance was varied during the environmental temperature changes because of the transformation of the P(NIPAAm) structure. We provide a simple and novel sensing mechanism on the circuit electrode to exhibit the sensor’s application value to humans.

## 2. Experimental Details

### 2.1. Preparation of Circular Electrode and Glass

**Figure 1:**
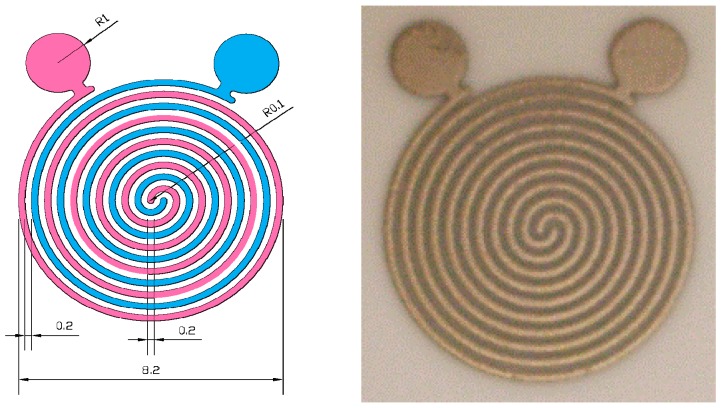
The structure is circular electrode (size: 1 cm × 1 cm).

The structure of circular electrode used is shown in Figure 1. It was made by screen-printing silver paste on the alumina substrate and then sintering. This kind of electrode has advantages of low point discharge and large effective area, so it promotes measurement sensitivity and decreases the loss of signal from point discharge.

The slide glass and circular electrode were cut into size of 1 mm × 1 mm. The substrate electrodes were then cleaned ultrasonically in 95% ethyl alcohol solvent and distilled water for 15 min and dried in desiccators to remove the surface contaminants and organic matters. This would ensure a good adhesion between the substrate and the deposited films [18,19,20].

**Figure 2:**
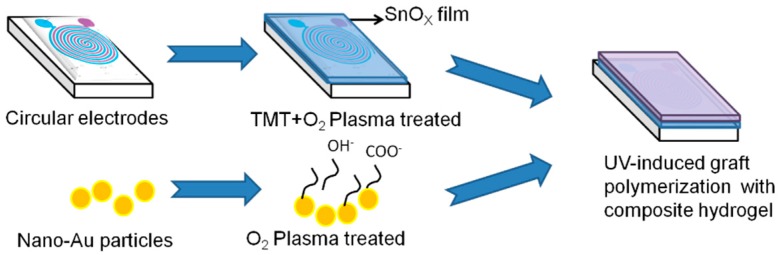
Flow chart in this study.

This study used radio frequency (RF) oxygen plasma treatment as a process for the polymeric substance to be grafted onto the substrate. A new type temperature sensor was successfully produced by UV-induced grafting polymerization of NIPAAm monomer onto AuNPs and then immobilization of grafted AuNPs onto the electrode substrate. It combines the advantages of electrical conductivity of AuNPs with temperature sensibility of NIPAAm hydrogel. This new composite device is small in dimension, fast in response, lower cost in fabrication, high stability in practical usage, and can improve the disadvantage of common resistance type temperature sensors. The experimental flow chart of this study is shown in Figure 2.

### 2.2. Plasma Pre-Treatment

AuNPs were obtained by reducing trisodium citrate and hydrogen tetrachloroaurate(III) tetrahydrate (chloroauric acid) and modifying with 11-mercaptoundecanoic acid (MUA) by the self-assembly monolayers (SAM) [21]. The AuNPs were subjected to O_2_ plasma pretreatment to form peroxide groups on the surface. The plasma polymerization system mainly comprises a bell-jar reaction chamber and 13.56 MHz radio frequency generators (model OEM-6 POWER SYSTEMS, Eni Co., Rome, Italy) [22]. The AuNPs (concentration: 1.5 mL, 3.0 mL and 4.5 mL dried in 40 °C) were first subjected to oxygen plasma treatment to activate groups (OH^−^, COO^−^) on the particle surface. The O_2_ plasma treatment was operated at 100 W, 40 mTorr for 1 min. In oxygen plasma treatment, two processes may occur: etching of the polymer surface through the reactions of atomic oxygen with the surface carbon atoms to produce volatile reaction products, and the formation of oxygen functional groups at the surface through the reactions between the active species from the plasma and the surface. The balance of these two processes depends on the operation parameters of a given experiment.

Prior to immobilization by AuNPs to the circular electrode, the circular electrode was modified with TMT and O_2_ plasma to enhance conductivity by forming a layer of SnO*x*C*y* film on the surface. The chamber was pumped down to a base pressure of 30 mTorr before introducing mixtures of TMT (Sn(CH_3_)_4_), purity > 99%, vacuum degassed) and oxygen gas (purity > 99%). Deposition was performed at an input power of 100 W, deposition time of 10 min, and TMT/O_2_ ratio of 40:40 in mTorr with the rate flow of O_2_ about 40 sccm.

### 2.3. Post Treatment by Photo UV-Induced Grafting Polymerization

The schematic diagram of UV-light system is (Sunshine Inc., Taipei, Taiwan) [23]. The electrode substrates after plasma treatment were immersed into the different AuNPs concentration (1.5 mL, 3.0 mL and 4.5 mL) and NIPAAm monomer mixture solution in Pyrex glass utensil. The glass utensil was sealed and then irradiated with UV light (wavelength = 365 nm^−1^) at 2000 W for 15 min to induce grafting polymerization. After UV polymerization, the NIPAAm became p-NIPAAm hydrogel with the mixture of AuNPs. The samples were then washed in distilled water. The thickness of NIPAAm-AuNPs film has been controlled in 1 mm.

### 2.4. Characterization

#### 2.4.1. Surface Contact Angles

The water contact angles (WCA) of the untreated and the subsequent deposited films were measured by the sessile drop (0.2–0.3 mL) method with distilled water by a syringe and observed by CCD at room temperature (25.0 °C) (Goni-meter type G-1 made by ERMA Optical Works Co., Ltd., Tokyo, Japan). The drop image was recorded by a video camera. The measured WCA value was the average of three measurements.

#### 2.4.2. Morphology

The surface morphology of plasma deposited films was observation of scanning electron microscope (JSM-5600, JEQL, Tokyo, Japan). The samples were placed on aluminum or copper holder and sputtering coated with a thin layer of gold (coating 90 s) to improve the electrical conductivity.

#### 2.4.3. UV-VIS Spectra

Ultraviolet-visible spectroscopy (Jasco V-560, 300 nm–800 nm, Jasco, Tokyo, Japan) refers to absorption spectroscopy in the ultraviolet-visible spectral region. The absorption or reflectance in the visible range directly affects the perceived color of the chemicals involved. In this study, calibration was determined from the relation between AuNPs concentrations and UV-vis specific wavelength (525.5 nm) intensity. Through this way, the distribution of AuNPs in NIPAAm solution could be obtained with high accuracy.

#### 2.4.4. Resistance Measurement

Keithey 2000 measured the resistance of electrode. The output/input terminals of electrodes were first connected to the equipment and kept in the dry air until the signal was stable. The electrode was then soaked in the deionized water in a heat-resistance glass container, and the deionized water was heated to the temperature of 45.0 °C. The resistance was recorded per 15 s to a precision of one degree.

## 3. Results and Discussion

### 3.1. Resistance and Surface Contact Angles

The values of circular electrode resistance after different treatments are listed in Table 1. The resistance of the pure AuNP solution was 11.9 kΩ, while that of the TMT + O_2_–treated electrode was 20.8 kΩ. Because of the low conductivity of the NIPAAm hydrogel, the resistance increased to 130 kΩ after the immobilization of NIPAAm hydrogel on the circular electrode. From Table 1, it is clearly indicated that with the increase in the treated AuNPs, the resistance of the composite hydrogel decreased (from 130 kΩ to 60.4 kΩ) due to the excellent conductivity of the AuNPs. The concentration in the composite hydrogel improved the conductivity of the circular electrode.

The values of the water contact angle (WCA) of the electrode substrate after each treatment are listed in Table 2. The WCA of the glass substrate after the TMT + O_2_ treatment decreased from the untreated substrate (40.1°) to less than 10°, exhibiting a hydrophilic property. The WCA increased to 41.0° with the immobilization of the NIPAAm hydrogel on the substrate surface. The time for completing the absorption of water by the NIPAAm hydrogel took about 100 s. After the absorption by distilled water, the measured WCA decreased again to less than 10°. This illustrates that the absorption time of the hydrogel may affect the sensitivity and stability of the composite hydrogel sensor. The effect of the soaking time on the resistance of the electrode will be discussed later in this work.

### 3.2. Environmental Test

**Figure 3:**
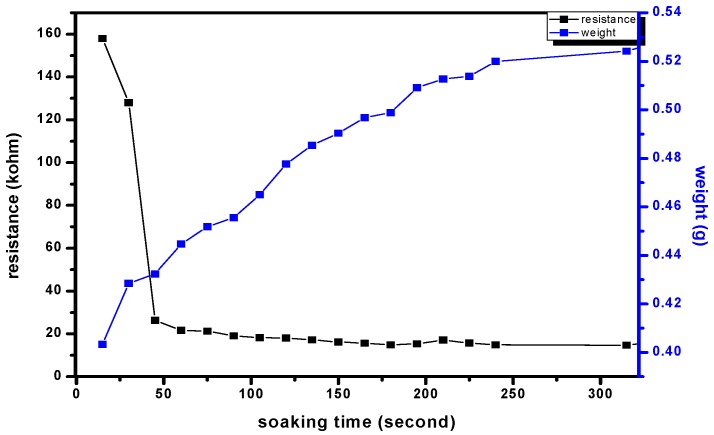
The variations are electrode resistance as well as hydrogel mass with soaking time.

The variations of the electrode resistance as well as the hydrogel mass with the soaking time at 25 °C are shown in Figure 3. At the initial 100 s, the NIPAAm hydrogel absorbed most of the deionized water, therefore exhibiting a drastic resistance drop. However, with a further increased soaking time, the resistance decreased at a slow rate as reflected from the small slope, indicating that hydrogel becomes saturated when the soaking period is more than 100 s; thus, there was no significant variation of the resistance with the soaking time.

**Figure 4:**
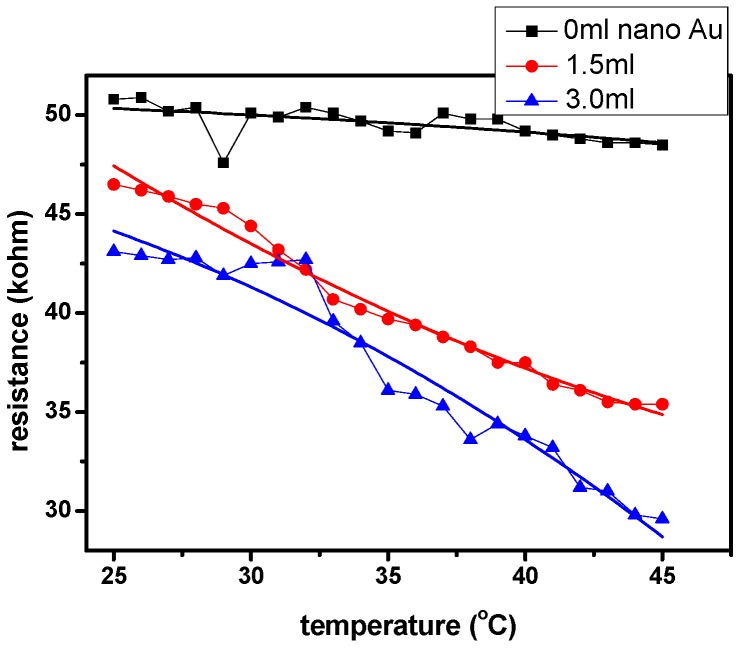
The variations are of resistance with temperature for two samples.

**Figure 5:**
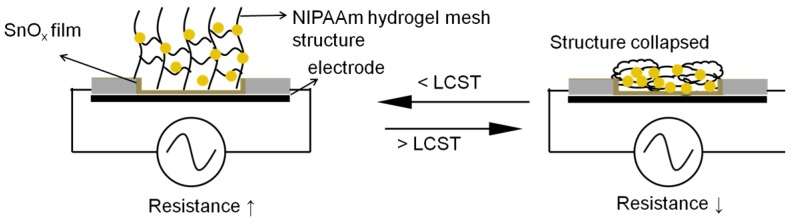
Morphological changes in NIPAAm hydrogel structure.

Figure 4 shows trend of the resistance with the temperature for specimens with different amounts of AuNPs. Being soaked in water for 100 s, these samples were slowly heated from 25 °C to 45 °C (rate = 1 °C/1 min). The resistance was recorded accordingly as the temperature increased by each degree. As seen in Figure 4, the resistance dropped linearly with the increased temperature. The fitted curve slopes of specimens with 0 mL, 1.5 mL and 3.0 mL AuNPs are −0.84, −2.30 and −1.22, respectively. It should be pointed out that when the temperature is higher than 31 °C, the composite hydrogel becomes a globular collapsed structure and causes the AuNPs to contact the electrode surface. When the temperature is lower than 31 °C, the structure of the NIPAAm becomes unfolded. The morphological changes in the NIPAAm hydrogel structure are illustrated in Figure 5.

### 3.3. Scanning Electron Microscope (SEM) Morphology

**Figure 6:**
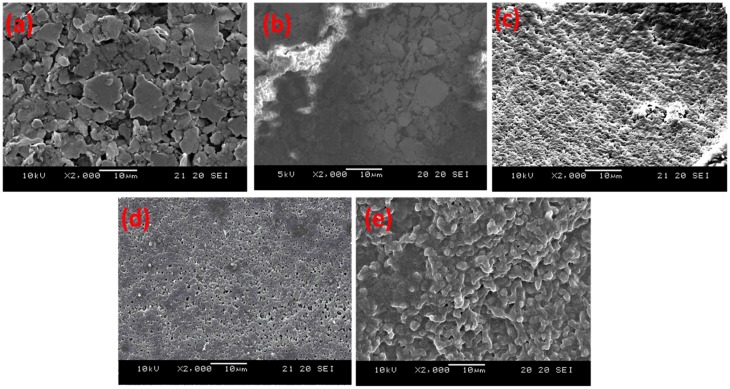
SEM morphologies of (**a**) untreated sample, and composite gels containing (**b**) 0 mL; (**c**) 1.5 mL; (**d**) 3.0 mL; and (**e**) 4.5 mL AuNPs (2000×).

The scanning electron microscope (SEM) morphologies of the samples after various treatments are shown in Figure 6. Figure 6a shows the surface of the untreated electrode. After grafting with the NIPAAm hydrogel only, the electrode surface was covered with a homogeneous hydrogel layer and the surface roughness was small (Figure 6b). Figure 6c–e demonstrate the electrode surface with grafted hydrogels in amounts of 1.5 mL, 3.0 mL and 4.5 mL AuNPs, respectively. The surfaces in these figures are rough with a large number of pores as compared to the image in Figure 6b. The pore structure is believed to provide a larger contact area and thus can enhance the sensitivity of the sensor. In particualr, samples with 1.5 mL and 3 mL AuNPs exhibited the largest number of pores per area on the surface.

### 3.4. UV-Vis Spectra

**Figure 7:**
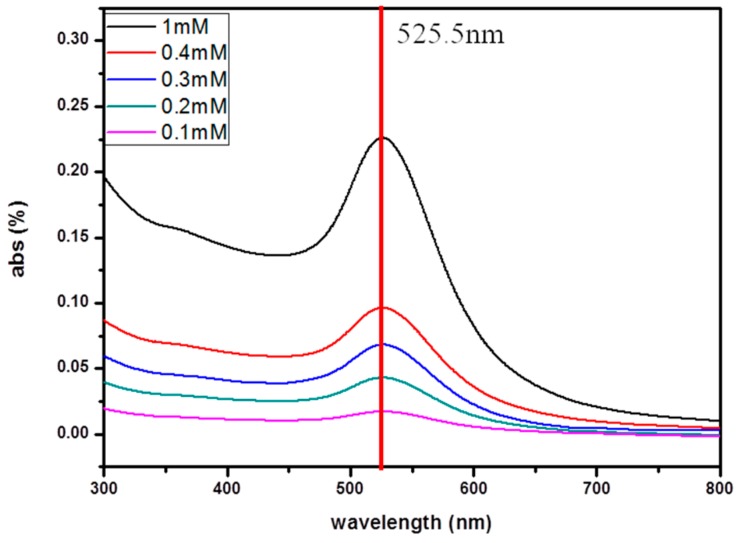
UV-vis spectra of AuNPs in different concentrations and the corresponding calibration curves.

To evaluate the distribution of AuNPs in the NIPAAm solution, UV-vis was used to detect the intensity of the specific absorption peak at 525.5 nm. Figure 7 shows the UV-vis spectra of AuNPs of different concentrations as well as the calibration curve. The slope of the calibration curve is 0.99, indicating that particles are uniformly distributed in the NIPAAm solution. From the calibration curve and ZETA potential analysis, the concentration of AuNPs was found to be 1.07 × 10^13^ (particles/mL) and the particles’ average size was 15.6 ± 2.7 nm.

### 3.5. Response and Recovery Test

**Figure 8:**
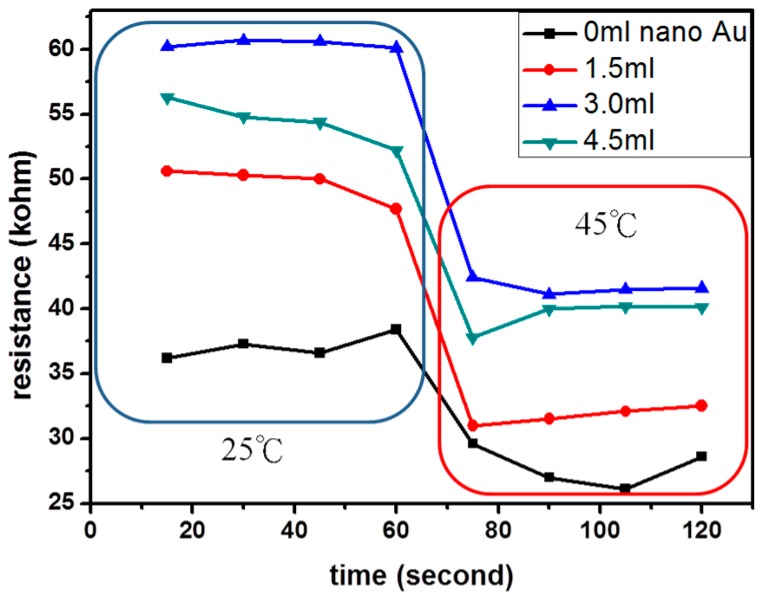
Sensor response of sensor devices containing Black—0 mL; Red—1.5 mL; Blue—3.0 mL; and Green—4.5 mL AuNPs between 25 °C and 45 °C over a period of 2 min.

The response of composite sensors is estimated by measuring the variation of resistance with time at temperatures between 25 °C and 45 °C over a period of 2 min, and the results of the composite sensors containing 1.5 mL, 3.0 mL and 4.5 mL of AuNPs are shown in Figure 8. This Figure indicates that the addition of 1.5 mL AuNPs to the hydrogel was the best with a resistance decrease from 45.5 kΩ to 35.4 kΩ in 1 s and had the highest sensitivity of 0.39. Furthermore, the result shows that both the response time and recovery time were short. Compared to the control sample, the NIPAAm hydrogels with added AuNPs enhanced the sensitivity and stability of temperature detection.

## 4. Summary

In this study, nano-Au particles with a NIPAAm hydrogel were successfully grafted on the electrode surface from a composite hydrogel for temperature sensing. Using the ambient environment resistance test to measure the resistance variation of the sensor, it was found that the resistance dropped linearly with the increased temperature due to the collapse of the NIPAAm hydrogel to a globular shape. When the environment temperature was below 31 °C, the NIPAAm hydrogel swelled up and caused an increase in the resistance. This change in the NIPAAm structure is a reversible reaction so the sensor can be reusable. From the calibration curve and ZETA potential analysis, the concentration of AuNPs was found to be 1.07 × 10^13^ (particles/mL) and the particles’ average size was 15.6 ± 2.7 nm. The stability for samples with the addition of 1.5 mL AuNPs was the best with a sensitivity of 0.39.

## Figures and Tables

**Table 1:** The values are the circular electrode resistance after different treatments.

Different Treatment	AuNPs Solution	Treatment A	Treatment B	Treatment C	Treatment D	Treatment E
Resistance (KΩ)	11.9 ± 2.3	20.8 ± 2.2	130 ± 5.5	59.2 ± 3.4	61.5 ± 4.9	60.6 ± 2.9

Treatment A—TMT + O_2_ plasma treatment; treatment B—Hydrogel with 0 mL AuNPs; treatment C—Hydrogel with 1.5 mL AuNPs; treatment D—Hydrogel with 3.0 mL AuNPs; and treatment E—Hydrogel with 4.5 mL AuNPs.

**Table 2:** Water contact angles of electrode substrate after different treatments.

Different Treatment	Untreated	Treatment A	Treatment B	Treatment C	Treatment D	Treatment E
θ_H_2_O_	40.1°	<10°	41.0°	31.5°	43.2°	43.1°

Treatment A—TMT + O_2_ plasma treatment; treatment B—Hydrogel with 0 mL AuNPs, treatment C—Hydrogel with 1.5 mL AuNPs; treatment D—Hydrogel with 3.0 mL AuNPs; and treatment E—Hydrogel with 4.5 mL AuNPs.
